# Primary Meningococcal Pericarditis Due to Neisseria meningitidis: A Case Report

**DOI:** 10.7759/cureus.89395

**Published:** 2025-08-05

**Authors:** Areeba Hasan, Saif M Khan, Lulu Azeez

**Affiliations:** 1 Department of Medicine, Tbilisi State Medical University, Tbilisi, GEO; 2 Internal Medicine, Aster Hospital, Dubai, ARE

**Keywords:** bacterial pericarditis, meningococcemia, neisseria meningitidis, primary meningococcal pericarditis, purulent pericarditis

## Abstract

This case report describes primary meningococcal pericarditis (PMP), a rare and potentially life-threatening form of *Neisseria meningitidis* infection that may mimic idiopathic or viral pericarditis, particularly in the absence of classic signs of meningococcemia or meningitis. PMP accounts for a small proportion of meningococcal pericarditis cases. Early in its course, PMP can present without systemic features or hemodynamic instability, complicating timely recognition. Clinicians should maintain a high index of suspicion when encountering mild leukocytosis with a left shift or bandemia in patients with presumed uncomplicated pericarditis, as this may represent an early indicator of invasive meningococcal disease requiring prompt investigation and treatment.

## Introduction

Pericarditis is a rare but serious complication of meningococcal infection, first described by Herrick in 1918. The incidence of meningococcal pericarditis across all age groups has been reported to range between 4% and 19% of invasive meningococcal disease cases; however, this range includes both primary and reactive forms and does not reflect the rarity of isolated PMP [1].

Although pericarditis itself is a relatively common clinical entity, its etiologies are diverse and broadly divided into infectious and non-infectious causes. Viral agents, particularly Coxsackie viruses and other enteroviruses, represent the most frequent infectious causes. Bacterial pericarditis, while less common, is clinically significant and associated with high morbidity. Viral agents, particularly Coxsackie viruses and other enteroviruses, represent the most frequent infectious causes. Bacterial causes, although less common, are clinically significant and include pathogens such as *Staphylococcus aureus*, *Streptococcus pneumoniae*, and *Neisseria meningitidis* [2]. Among bacterial etiologies, *N. meningitidis*, particularly Group C strains, is a recognized though infrequent cause of primary pericarditis, accounting for approximately 6% of non-tuberculous bacterial pericarditis cases [3].

Meningococcal pericarditis can occur as either primary meningococcal pericarditis (PMP), involving direct bacterial invasion of the pericardium without systemic meningococcemia, or as reactive pericarditis secondary to disseminated meningococcal infection, where sterile pericardial inflammation develops as part of an immune response. Recognizing this distinction is crucial, as primary forms often lack classical features of meningitis or sepsis and can mimic benign viral pericarditis, posing significant diagnostic challenges.

Despite its rarity, bacterial pericarditis is associated with significant morbidity and mortality. The reported mortality rate is approximately 40% even with appropriate treatment and as high as 85% in untreated cases, underscoring the importance of early recognition and aggressive management [4].

## Case presentation

A 52-year-old male with type 2 diabetes presented to the emergency department (ED) with a two-day history of chest pain and epigastric discomfort. Initial laboratory investigations revealed a troponin level of 8 ng/L, total leukocyte count (TLC) of 17 × 10⁹/L, hemoglobin of 17 g/dL, platelet count of 128 × 10⁹/L, neutrophils at 89%, and a markedly elevated C-reactive protein (CRP) level of 392 mg/L. The patient denied symptoms of fever, cough, vomiting, diarrhea, or signs of meningial infection or nuchal rigidity, and he was discharged with symptomatic treatment.

Two days after discharge, the patient re-presented with worsening symptoms, now including elevated blood glucose levels and mild metabolic acidosis. A 2D echo revealed pericardial effusion, and a clinical diagnosis of pericarditis was made. A provisional diagnosis of diabetic ketoacidosis was made based on clinical features and mild metabolic acidosis. He was started on an insulin infusion. The patient was then admitted to the ICU under the care of a cardiologist.

**Figure 1:**
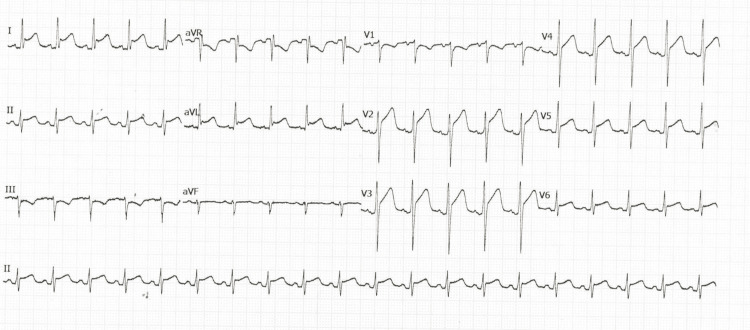
Electrocardiogram on admission showing sinus rhythm with diffuse upward ST-segment elevations consistent with pericarditis.

On re-admission, the patient was normotensive and maintained an oxygen saturation of 92% on room air. Capillary blood glucose was 268 mg/dL while on IV insulin infusion. No signs of peripheral edema or deep vein thrombosis were noted. Electrocardiography (ECG) demonstrated widespread concave-upward ST-segment elevations (Figure 1).

Chest X-ray showed cardiomegaly. Inflammatory markers remained elevated. A broad differential diagnosis was initially considered, including HIV, Coxsackie virus, EBV, parvovirus, and CMV, suggesting viral pericarditis. The patient was managed with colchicine, nonsteroidal anti-inflammatory drugs (NSAIDs), and IV ceftriaxone. Sepsis was suspected given the markedly elevated inflammatory markers, including a total leukocyte count of 19.6 × 10⁹/L, ESR of 75 mm/h, CRP of 451 mg/L, and procalcitonin at 28.4 ng/mL. Glycemic control was poor, with an HbA1c of 9.7%. The peripheral smear demonstrated neutrophilia with mild thrombocytopenia. 

He also reported swelling in his right wrist and left ankle. The diagnosis of reactive arthritis was based on joint swelling in the context of systemic inflammation and meningococcal infection, supported by elevated inflammatory markers and the temporal relationship to infection onset. Joint fluid analysis was not performed; thus, septic arthritis could not be definitively excluded. He was started on NSAIDs.

**Figure 2:**
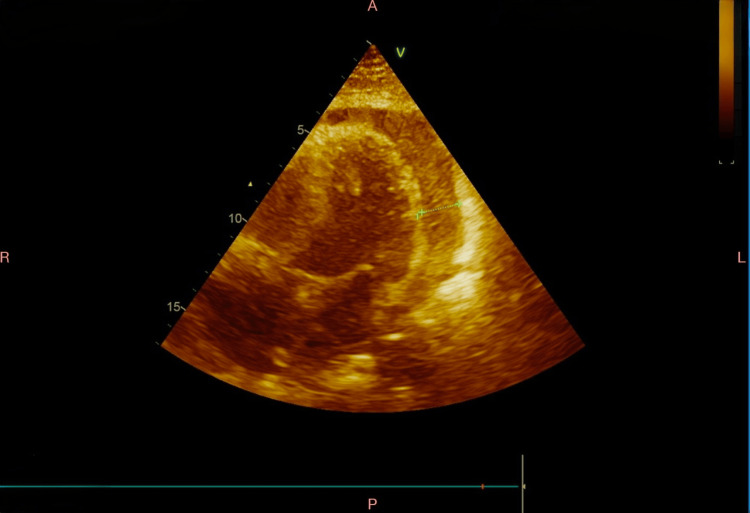
Two-dimensional echocardiogram demonstrating moderate pericardial effusion.

Blood cultures later grew gram-variable cocci, prompting escalation of antibiotics to intravenous ceftriaxone 2 g twice daily. Echocardiography revealed normal left ventricular systolic function (LVEF 55%) but a moderate-sized pericardial effusion posterior to the LV/apex (9-11 mm) and behind the RA/RV (6-8 mm), with internal echoes suggestive of purulent material (Figure 2). The IVC was 1.9 cm with reduced collapse. The effusion was minimal anteriorly, and pericardiocentesis was not considered safe due to the location. Surgical drainage was recommended for the purulent collection.

The patient subsequently reported worsening chest pain and breathing difficulty, with bilateral crepitations on lung examination. Effusion is now worse and is circumferential with mild early diastolic collapse of the right ventricle (RV). Based on these findings, the patient was transferred immediately to surgery for further management. Upon arrival, the patient remained symptomatic but stable. Given the early echocardiographic signs of tamponade, an emergency left thoracotomy to create a pericardial window was performed. A tense pericardial collection of about 200 mL and fluid was drained along with debris of pericardial infection, leading to significant symptomatic improvement. A pericardial drain was left in place. 

Blood culture at this point was positive for *Neisseria meningitidis*. Pericardial fluid samples were sent for analysis, and antibiotics were continued for potential meningococcal sepsis pending further results. Additional infectious screenings, including for hepatitis B, hepatitis C, and HIV, were negative. 

Poor glycemic control in the setting of type 2 diabetes mellitus contributed to the development of diabetic ketoacidosis, precipitated by systemic infection. Moderate left ventricular systolic dysfunction developed postoperatively as documented on serial echocardiography, with features consistent with stress cardiomyopathy, diagnosed based on transient LV dysfunction without evidence of obstructive coronary disease. The patient was successfully extubated following stabilization, with a progressive decline in C-reactive protein indicating resolution of the inflammatory response.

**Figure 3:**
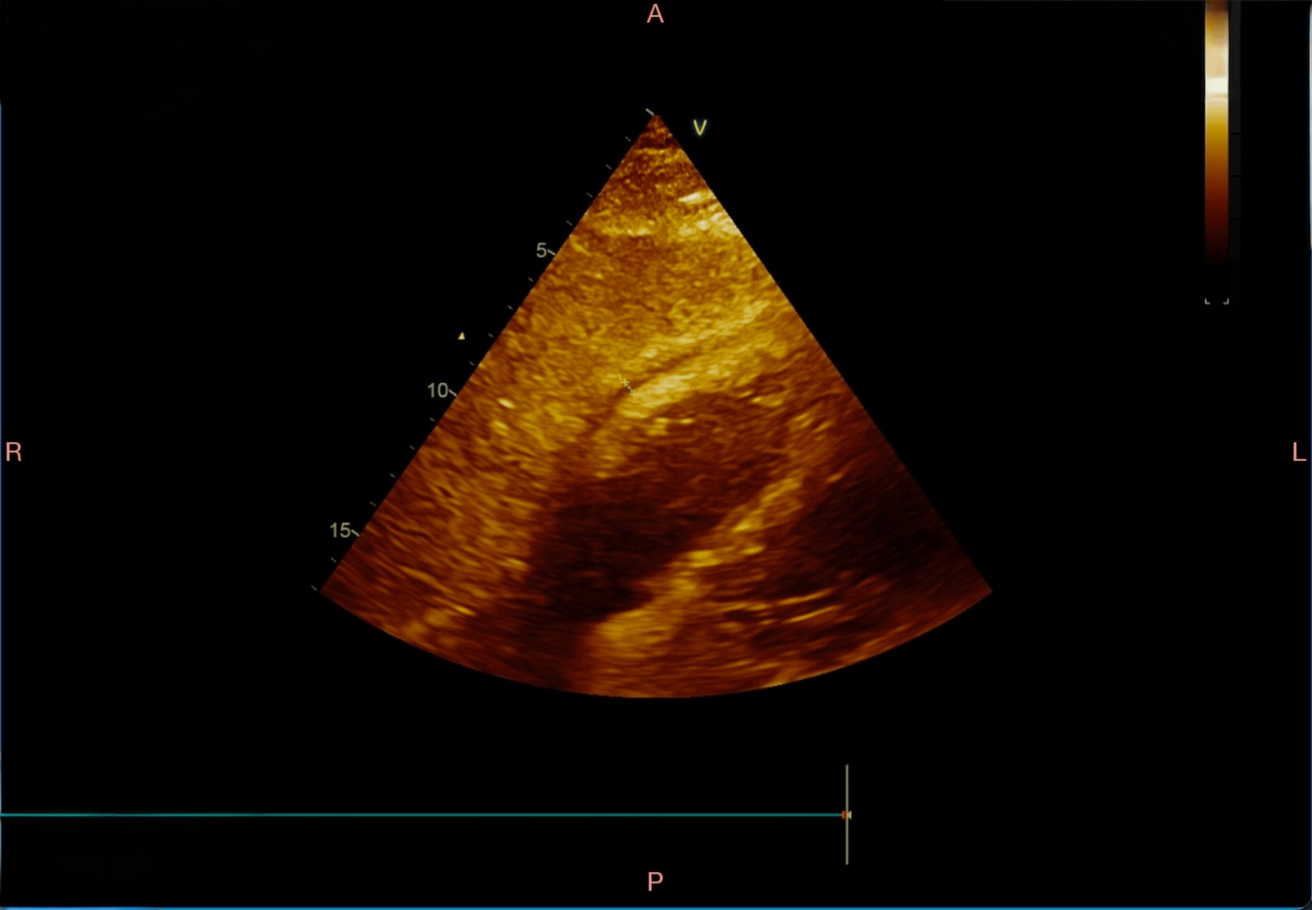
Follow-up echocardiogram illustrating improvement and reduction of pericardial effusion.

Serial echocardiograms showed improvement in the pericardial effusion (Figure 3), with no further signs of tamponade. The pericardial drain was removed three days after insertion. Pericardial fluid cultures showed no growth. The patient was then shifted to ward nine days after initial presentation, with ongoing IV antibiotics, and was discharged 14 days after initial presentation with a short course of cefixime for seven days.

## Discussion

This case highlights an uncommon manifestation of *N. meningitidis* as purulent pericarditis in an adult patient with poorly controlled type 2 diabetes mellitus, further complicated by diabetic ketoacidosis and stress cardiomyopathy. Invasive meningococcal disease often presents as meningitis or the frequently fatal acute meningococcemia. However, *N. meningitidis *can cause a range of other less recognized but significant presentations, including pericarditis, conjunctivitis, panophthalmitis, pneumonia, urogenital infections, and arthritis [5].

Meningococcal pericarditis is typically classified into three types: pericarditis as part of disseminated meningococcal disease, primary (isolated) meningococcal pericarditis, and reactive meningococcal pericarditis, which manifests as serous, sterile pericardial effusion following successful antibiotic therapy in the post-infectious phase [6,7].

Meningococci account for 5.9% of purulent pericarditis cases, ranking fourth after *Staphylococcus*, *Streptococcus pneumoniae*, and *Streptococcus* species [7]. Disseminated meningococcal pericarditis (DMP) generally occurs early in the infection course due to hematogenous spread to the pericardium. By contrast, immune reactive pericarditis (IRP), which arises later, is a hypersensitivity response causing sterile inflammation of the pericardium, which responds better to anti-inflammatory agents than antibiotics [8,9]. Primary meningococcal pericarditis (PMP) is defined as purulent pericarditis caused by *N. meningitidis* in the absence of meningococcemia, meningitis, or other systemic involvement [10]. It is described as “purulent and culture-positive, yet without evidence of meningeal or systemic clinical features” [3]. PMP is exceptionally rare, often difficult to identify at onset, and frequently associated with rapid progression to complications. The isolation of meningococci from either blood or pericardial fluid in all reported cases and the finding of purulent fluid on initial aspiration strongly suggests early pericardial invasion by the organism. While hematogenous spread from meningococcemia is the most probable route, direct extension from pleuropulmonary tissue (similar to pneumococcal pericarditis) is also conceivable. In our patient, no radiological evidence of contiguous pleuropulmonary infection was identified, making hematogenous seeding the more plausible mechanism [11]. Common presenting symptoms include chest pain, fever, and dyspnea. Patients typically report a two-day history of such symptoms before a cardiac etiology is considered, mirroring our case, where the patient initially presented with chest pain and epigastric discomfort [10]. It is important to note that patients with PMP may appear only mildly ill at presentation, and symptoms can be misinterpreted as uncomplicated viral pericarditis. In our case, the patient’s atypical presentation with epigastric discomfort and chest pain underscores this diagnostic challenge.

The most affected patients are young adults with equal distribution across genders. Notably, underlying immunosuppression or chronic illness did not appear to be contributing risk factors [10]. Most patients had leukocytosis on presentation. Chest X-rays commonly showed an enlarged cardiac silhouette, as observed in our patient [12]. Electrocardiograms typically demonstrated diffuse ST-segment elevation consistent with pericarditis. Echocardiography usually revealed pericardial effusion or cardiac tamponade, with tamponade features reported in 62-88% of cases. While pericardiocentesis remains the first-line intervention for diagnostic and therapeutic relief of pericardial effusion, a surgical pericardial window may be preferred in purulent pericarditis due to its superior drainage efficacy and reduced risk of re-accumulation. In our case, the choice of surgical window was guided by the purulent nature of the fluid and the clinical instability suggestive of tamponade physiology. Blood cultures were positive in 44-75% of reported cases, which was also observed here [10,12].

Arthritis is a recognized complication in 2-10% of meningococcal infections. It most commonly arises during the convalescent phase as a sterile effusion involving large joints, representing a reactive arthritis secondary to an intense immune response. In contrast, direct bacterial invasion of the synovium may occur during the acute phase of infection, as in the present case, or the setting of chronic meningococcaemia [5].

The recommended treatment for meningococcal infection is benzylpenicillin or third-generation cephalosporins like cefotaxime or ceftriaxone. In the report by Morgan et al., initial treatment with benzylpenicillin failed to resolve the infection, but the third-generation cephalosporin provided was effective, likely due to its greater sensitivity and longer half-life (approximately one hour versus penicillin’s 30-minute half-life) [9]. In our case, the patient was started on 2 g of cefotaxime, a third-generation cephalosporin, intravenously every 12 hours, which led to a prompt clinical improvement. 

Although symptom resolution has been reported within three to six days with treatment [5], this refers primarily to relief of pericardial symptoms and may not reflect full clinical recovery.

Public health measures are a critical component of managing confirmed meningococcal infections. Upon diagnosis, mandatory notification to local health authorities is required to enable timely epidemiological surveillance and outbreak prevention. Close contacts, including household members and healthcare workers with unprotected exposure, must receive chemoprophylaxis to curb the risk of secondary spread.

## Conclusions

PMP is a rare and rapidly progressive condition that warrants inclusion in the differential diagnosis of pericarditis, particularly when presenting with atypical or nonspecific symptoms such as epigastric discomfort. Its early presentation may mimic benign viral pericarditis, but clinical features like leukocytosis, persistent fever, or failure to respond to anti-inflammatory therapy should raise suspicion for a bacterial cause. Blood cultures and echocardiography are critical diagnostic tools that enable early identification of PMP and assessment of hemodynamic compromise.

Timely intervention with targeted antibiotic therapy and pericardial drainage is essential to reduce morbidity. In cases where pericardiocentesis is not feasible or insufficient, surgical drainage should be promptly considered to prevent progression to cardiac tamponade. Patients with underlying conditions such as diabetes mellitus may have atypical presentations or worse outcomes, underscoring the importance of heightened clinical vigilance in such populations.
